# CD300b regulates intestinal inflammation and promotes repair in colitis

**DOI:** 10.3389/fimmu.2023.1050245

**Published:** 2023-03-22

**Authors:** Shmuel Avlas, Hala Kassis, Michal Itan, Hadar Reichman, Avishay Dolitzky, Inbal Hazut, Sharon Grisaru-Tal, Yaara Gordon, Ilan Tsarfaty, Danielle Karo-Atar, Perri Rozenberg, Almog Bitton, Ariel Munitz

**Affiliations:** Department of Microbiology and Clinical Immunology, Faculty of Medicine, Tel Aviv University, Tel Aviv, Israel

**Keywords:** CD300, colitis, inflammation, macrophages, soluble CD300b

## Abstract

Chronic inflammation is a hallmark charataristic of various inflammatory diseases including inflammatory bowel disease. Subsequently, current therapeutic approaches target immune-mediated pathways as means for therapeutic intervention and promotion of mucosal healing and repair. Emerging data demonstrate important roles for CD300 receptor family members in settings of innate immunity as well as in allergic and autoimmune diseases. One of the main pathways mediating the activities of CD300 family members is *via* promotion of resolution through interactions with ligands expressed by viruses, bacteria, or dead cells (e.g., phospholipids such as PtdSer and/or ceramide). We have recently shown that the expression of CD300a, CD300b and CD300f were elevated in patients with IBD and that CD300f (but not CD300a) regulates colonic inflammation in response to dextran sodium sulphate (DSS)-induced colitis. Whether CD300b has a role in colitis or mucosal healing is largely unknown. Herein, we demonstrate a central and distinct role for CD300b in colonic inflammation and subsequent repair. We show that *Cd300b^-/-^* mice display defects in mucosal healing upon cessation of DSS treatment. *Cd300b^-/-^* mice display increased weight loss and disease activity index, which is accompanied by increased colonic histopathology, increased infiltration of inflammatory cells and expression of multiple pro-inflammatory upon cessation of DSS cytokines. Furthermore, we demonstrate that soluble CD300b (sCD300b) is increased in the colons of DSS-treated mice and establish that CD300b can bind mouse and human epithelial cells. Finally, we show that CD300b decreases epithelial EpCAM expression, promotes epithelial cell motility and wound healing. These data highlight a key role for CD300b in colonic inflammation and repair processes and suggest that CD300b may be a future therapeutic target in inflammatory GI diseases.

## Introduction

Inflammatory bowel diseases (IBDs) including ulcerative colitis (UC) and Crohn’s disease (CD) are chronic, relapsing inflammatory diseases causing substantial morbidity. Chronic inflammation, which accompanies IBD is characterized by the infiltration of various immune cells and increased expression of multiple proinflammatory cytokines including IL-6, TNF-α IL-12, IL-23, and IFN-γ (1). Subsequently, current therapeutic approaches mainly target immune-mediated pathways as means for therapeutic intervention (2). These therapeutic strategies include amino-salicylates, antibiotics, glucocorticoids and immunomodulatory agents. Ultimately, dampening inflammation leads to disease remission, which is accompanied by significant mucosal healing (3). Thus, defining pathways, which mediate colonic inflammation could lead to new therapeutic targets in IBD.

The CD300 receptor family is composed of several (i.e., eight receptors in human and nine in mice) type I transmembrane glycoproteins with a single IgV-like extracellular domain and either an immunoreceptor tyrosine-based inhibitory or activation motifs (ITIM and ITAM, respectively) in their cytoplasmic tail (4). The genetic loci of CD300-family members (mapping to human chromosome 17q22-25 and chromosome 11 in mice) are one of only a few loci in the genome that is under strong positive evolutionary selection likely indicating the importance of this receptor family in immune regulation (5, 6). CD300 receptors can bind and recognize several ligands including phosphatidylserine (PdtSer) (7–9), phosphatidylethanolamine (7), ceramide (10, 11), and lipopolysaccharide (LPS) (12) all of which are present in settings that are accompanied with inflammation and cell death.

We have recently shown that the expression of CD300a, CD300b and CD300f were elevated in biopsies from pediatric UC patients and that their expression correlated with deep ulcerations (13). Furthermore, we have shown that CD300f (but not CD300a) regulates colonic inflammation in response to DSS-induced colitis (13). Whether CD300b has a role in colitis is largely unknown.

CD300b is predominantly expressed by myeloid cells (4), and through its short intracellular cytoplasmic domain it can interact with the immunoreceptor tyrosine-based activation motif (ITAM)-bearing adaptor molecule, DNAX-associated protein 12 (DAP12) upon recognition of PtdSer (9). Recent data demonstrate that CD300b can mediate efferocytosis *via* direct recognition of PtdSer (9). Indeed, binding of apoptotic cells to CD300b promotes the activation of the PI3K-Akt kinase pathway in macrophages, while silencing of CD300b expression diminishes PI3K-Akt kinase activation and impairs efferocytosis (9). In addition, CD300b was recently described as an integral part of the innate immune system. CD300b and its adaptor DAP12, associated with Toll-like receptor 4 (TLR4) upon binding to LPS. Association of CD300b to TLR-4, enhanced TLR4-dependent signaling, resulting in elevated pro-inflammatory cytokine production and decreased expression of IL-10 (12). In addition, artificial activation of CD300b by means of antibody cross-linking has been shown to promote the release of inflammatory cytokines from mast cells (14). While most of the studies examining the activities of CD300b focus on cell surface-expressed CD300b, it has been also shown that upon stimulation with LPS, the extracellular domain of CD300b can be cleaved to a solubule form (sCD300b), which can activate macrophages to amplify inflammation (15).

Herein we aimed to define the role of CD300b in colitis. We demonstrate that CD300b is required for mucosal healing following acute colonic inflammation. Our data further suggests that this phenomenon is mediated by binding of sCD300b to colonic epithelial cells. Collectively, these data uncover a new role for CD300b, which can be potentially translated into therapeutic interventions that promote mucosal healing.

## Methods

### Mice and experimental colitis

*Cd300b^-/-^* mice were kindly provided by Dr. Jiro kitura (Atopy Research Center, Tokyo, Japan). Mutant mouse strain was backcrossed to C57BL/6 mice for ten generations. Wild-type (WT) C57BL/6 mice were originally obtained from Harlan Laboratories (Rehovot, Israel) and grown in-house. In all experiments, age-, weight-, and sex-matched mice were used and housed under specific-pathogen-free conditions according to protocols approved by the Tel-Aviv University Institutional Animal Care Unit (Ethical approval #034_b14214_48, #034_b13792_20). C57BL/6 WT and *Cd300b^-/-^* mice were treated with 2% DSS (ICN biomedical Inc., Santa Ana, CA, average molecular weight of 41kDA) for five days in order to induce acute colonic inflammation. On day 5, the DSS was removed from the drinking water to initiate a repair stage. The mice were euthanized on day 11.

### Cell lines

All cell lines were obtained from ATCC, USA with the exception of MC38 which was obtained from ABM, USA. All human epithelial cell lines that were used in this study (i.e., HCT-15, HCT116, SW403, SW480, SW620) display epithelial cell morphology and were isolated from patients with colorectal cancer. MC38 cells are mouse epithelial cells that were derived from mouse colon carcinoma. RAW 264.7 cells are a monocyte/macrophage cell line that were isolated from the acytes of a mouse with an Abelson murine leukemia virus-induced tumor.

### zVAD-FMK treatment

C57BL/6 WT and *Cd300b^-/-^* mice were intraperitoneally injected with 0.1mg of the Pan-Caspase-inhibitor zVAD-FMK (Bachem, Bubendorf, Switzerland) dissolved in 10% DMSO (in Saline). 10% DMSO, which was dissolved in saline was used as control.

### Disease activity index scoring

The mice were evaluated for disease severity (or DAI) (16, 17) using the following clinical parameters: diarrhea (0=non,1=mild; soft stool, 2=moderate; liquid or/and soft stool stain surrounding the anus, 3=severe; massive liquid stool that stains the anus-side of the tail) and rectal bleeding (0=non; 1=mild; red blood spot on in the anus, 2=moderate; blood stains in the anus surroundings, 3=severe; blood stains in the anus surroundings and the tail) the maximum DAI score was 6.

### Enzymatic digestion of the colon

Mice were euthanized using isoflurane (100%, Abbott) and colon tissue were obtained and flushed with 2ml of calcium- and magnesium-free HBSS (biological industries). Colons were dissected longitudinally and shaked (250 r.p.m) in 5 ml calcium- and magnesium-free HBSS containing 5% fetal calf serum, 2mM EDTA, and 1mM DTT (dithiothreitol) for 40 min at 37°C in order to remove epithelial cells and intraepithelial leukocytes. Thereafter, the colonic tissue was vortexed and passed through a 100µm cell strainer (Corning, USA). The remaining tissue was incubated and shacked (250 r.p.m) with complete phosphate-buffered saline (PBS) +/+ (containing calcium and magnesium) supplemented with 5% fetal calf serum, 1 mg/ml^-1^ collagenase (Roche, Berlin, Germany) and 0.1 mg/ml^-1^ Dnase I (Sigma, Rehovot, Israel) for 40 min at 37°C. The cell suspension was further filtered through a 100µm cell strainer, washed in PBS and suspended in HBSS supplemented with bovine serum albumin (0.1%) + sodium azide (0.02%).

### Flow cytometry

CD300b expression and immunophenotyping experiments were conducted by flow cytometry as follows. In brief, colonic single-cell suspensions were blocked with 10% goat serum or anti-CD16/CD32 antibodies (clone 2.4G2) and stained with the following antibodies or combinations of these antibodies: anti-CD45-PE, anti-MHC-II-FITC, anti-Ly6c-PerCP-Cy5.5, anti-Ly6G-PE-Cy7, anti-CD11c-APC-eFlour780 (eBioscience, San Diego, CA), anti-CD11b-Brilliant violet 510 (Biolegend San Diego, CA), anti-CD300b (R&D Systems, Minneapolis, MN), goat-IgG (Peprotech, Rehovot, IL) and anti-goat-IgG-AlexaFlour647 (Jackson ImmunoResearch, West Grove, PA). In addition, colonic single-cell suspensions were stained with DAPI (Sigma, St. Louis, MO). Viable myeloid cells were identified as DAPI^-^/CD45^+^/CD11b^+^. Sub populations of myeloid cells were identified as follow (13): Neutrophils- MHC-II^-^/Ly6G^+^/Ly6c^-^; Monocytes- MHC-II^-^/, Ly6G^-^/Ly6c^+^; Eosinophils- MHC-II^-^/Ly6G^-^/Ly6c^-^/SSC^high^, Macrophages MHC-II^+^/Ly6G^+^/Ly6c^-^/Cd11c^low;^ dendritic cells MHC-II^-^/Ly6G^+^/Ly6c^-^/CD11c^high^. The expression of CD300b was determined as fold increase over isotype (CD300b median fluorescence intensity (MFI) divided by goat-IgG MFI) in each cell population.

Binding of sCD300b to epithelial cells was determined by incubating 10µg/ml of CD300b-Fc IgG1 fusion protein (Syd labs, lot 829880, Hopkinton, MA) or hIgG1 (Bioxcell, lot 659518N1, Lebanon, NH.) with colonic single-cell suspensions followed by staining with Alexaflour 647 conjugated with donkey anti hIgG. Thereafter, the cell suspension was stained for the myeloid cell population markers.

Raw 264.7, MC38, HCT15, HCT116, SW403, SW480 or SW620 cells (300X10^3^ cells) were incubated with 10µg/ml sCD300b (Biolegend, San Diego, CA) or hIgG (Bioxcell, lot 659518N1, Lebanon, NH.) for 30 min and then stained with Alexaflour647 conjugated donkey anti hIgG.

### Histopathology

Mice were euthanized at days 0, 5 and 11. Thereafter, the colons were obtained, fixed in 4% formaldehyde, embedded in paraffin and colonic tissue specimens were prepared (0.5μm). The slides were stained with H&E to assess the severity of colon pathology as previously described (18). TUNEL (Roche, TMR-red Kit, Rehovot, Israel or Abcam, HRP-DAB kit, Cambridge, UK) staining was used to assess apoptotic cell burden (19). Rabbit anti-mouse ki67 (Novus, CO, USA) was used on slides to quantify the proliferative epithelial cells. Alcian blue staining was applied on slides to detect goblet cells (GC). GC was quantified and the following score was given to each colon: 0= No epithelial tissue and no goblet cells, 1= up to 25% from epithelial layer are GC, 2 = 26%-50% from epithelial cells are GC, 3 = 51%-75% from epithelial cells are GC, 4 = 76%-100% from epithelial cells are GC.

### Immunofluorescence

A 22mm, rounded coverslip was placed at the bottom of each well in 6-well plate and 0.3X10^6^ MC38 were seeded and incubated overnight at 37°C. Subsequently, the cells were blocked (10% goat serum or anti-CD16/CD32 antibodies, clone 2.4G2) and incubated with 10µg/ml of CD300b-Fc IgG1 fusion protein (Biolegend, San Diego, CA) or hIgG1 (Bioxcell, lot 659518N1, Lebanon, NH.) followed by donkey anti hIgG conjugated to Alexaflour 647 (Jackson ImmunoResearch, West Grove, PA). Thereafter, the cells were fixed (4% paraformaldehyde) and stained with DAPI. At the end of this process, coverslips containing fixed and stained cell were flipped onto a glass slide containing a drop of mounting media and the cells were visualized and analyzed using fluorescence microscope. Binding of sCD300b to epithelial cells was determined by incubating 10µg/ml of CD300b-Fc IgG1 fusion protein or hIgG1 with colonic single-cell suspensions followed by staining with Alexaflour 647 conjugated with donkey anti hIgG. Thereafter, the cells were stained with FITC anti-mouse EpCAM antibody, fixed with 4% paraformaldehyde, stained with DAPI, and placed a glass slide using cytospin. Subsequently, the slides were mounted, covered, and analyzed using fluorescence microscope.

### Punch biopsies

The colons were flushed with PBS and opened along a longitudinal axis; 3mm^2^ punch biopsies were incubated for 24 h in RPMI supplemented with 10% fetal calf serum and antibiotics. Supernatants were collected for cytokine expression and saved in -80°C until used.

### Enzyme-linked immunosorbent assay

Cytokines and sCD300b were measured by ELISA according to manufacturer’s instructions using kits: IL-6, TNF-α, and IL-33 (R&D Systems, Minneapolis, MN), IL-10 (Biolegend San Diego, CA), CCL2, CXCL1, CCL11, EGF, and VEGF (peprotech, Rehovot, IL), sCD300b (OriGene Technologies Inc., Rockville, MD).

### Scratch assay

MC38 or SW480 cells (40X10^4^ cells) were seeded in 96 well plates in 100µl DMEM supplemented with10% FCS, 1% PS, and 1% Non-essential amino acids (NEAA) and incubated in 37^0^C, 5% CO2. sCD300b (10µg/ml, Biolegend, San Diego, CA) or hIgG (Bioxcell, lot 659518N1, Lebanon, NH.) were added for 12 hours or until the confluence was greater than 95%. Thereafter a scratch wound was made using 96-well wound-maker (ESSEN Bioscience Inc., Ann Arbor, MI). Subsequently, the cells were washed twice with PBS and 10µg/ml sCD300b or hIgG1 in 200µl Serum and NEAA free DMEM medium ware added. The wound healing were quantified using IncuCyte.

### Proliferation assay

MC38 or SW480 cells (40X10^4^ cells) were seeded in 96 well plates in 100µl DMEM supplemented with10% FCS, 1% PS, and 1% Non-essential amino acids (NEAA) and incubated in 37^0^C, 5% CO2. sCD300b (10µg/ml, Biolegend, San Diego, CA) or hIgG (Bioxcell, lot 659518N1, Lebanon, NH.) were added for 12 hours or until the confluence was greater than 95%. Thereafter, the cells were washed twice with PBS, and 10µg/ml sCD300b or hIgG1 in 200µl Serum and NEAA free DMEM medium were added. The amount of total cells per well were tracked during the cell culture and calculated using IncuCyte.

### Bone marrow derived macrophages

Bone marrow cells were collected by crushing the tibia and femur in PBS followed by cell filtering with cell strainer (70 µM; BD Bioscience, San Jose, CA). Total bone marrow cells were seeded in two 10 cm plates in 10 ml of DMEM medium (Gibco, Life Technologies, Carlsbad, CA) supplemented with 10% fetal bovine serum, 1mM sodium pyruvate, 1% penicillin-streptomycin (Biological Industries, Beit Haemek, IL) and 20 ng/ml macrophage-colony stimulating factor (Peprotech, Rehovot, Israel) for 7 days when 5 ml of supplemented medium were added for each plate at day 4. At day 7 macrophages purity (CD11b^+^/F4/80^+^ by flow cytometry) was higher than 95%.

### Generation of apoptotic cells

MC38 cells were grown to 95% confluence in 10cm plates using DMEM (Gibco, Life Technologies, Carlsbad, CA) supplemented with 10% fetal bovine serum, 1% penicillin-streptomycin (Biological Industries, Beit Haemek, IL). The cells were incubated with 5µl staurosphorine (Sigma-aldrich, St. Louis, Missouri) overnight. Apoptosis was determined by Annexin V-PI staining using flow cytometry.

### Efferocytosis assay

BMDMs were stained with carboxyfluorescein succinimidyl ester (CFSE, invitrogene, Carlsbad, CA) and incubated with Vybrant™ DiD-labeled (invitrogene, Carlsbad, CA) apoptotic MC38 cells (1:5 ratios, respectively) for 0, 15, 30, 60 min. The percentage of engulfed cells was determined by flow cytometry.

### Cell motility analysis

Blinded analysis of cell motility parameters was performed by tracking cellular movements during the scratch assay. Snap shots were taken every minute using IncuCyte. Thereafter, the images were collected, and single cell analysis of motility parameters was performed using IMARIS with the Imaris surface mode, as previously described (20). At least 100 cells were analyzed.

### RNA extraction and qPCR

Viable lymphoid (CD45^+^/CD11b^-^) and myeloid (CD45^+^/CD11b^+^) cells were sorted from colonic single cell suspensions of WT mice. Thereafter, RNA was extracted using the RNeasy Micro Kit (QIAGEN, Hilden, Germany) according to the manufacturer’s instructions. cDNA was synthesized using iScript cDNA kit (Bio-RAD, USA) and the expression of *Cd300b* (Fw primer: AATGACACGGACACTTACTGG, Rev primer: CATGTCTGTACTGCCGTCC) was calculated relative to *Hprt* (Fw primer: GTAATGATCAGTCAACGGGGGACA, Rev primer: CCAGCAAGCTTGCAACCTTAACCA) using iTaq Universal SYBER (BIO-RAD, USA).

### Statistical analysis

Data were analyzed by ANOVA followed by Tukey *post-hoc* test or Student’s *t*-test using GraphPad Prism 9 (San Diego, CA). Data are presented as mean ± SD, and values of *p* < 0.05 were considered statistically significant.

## Results

### Expression of sCD300b is increased during experimental colitis

**Figure 1:**
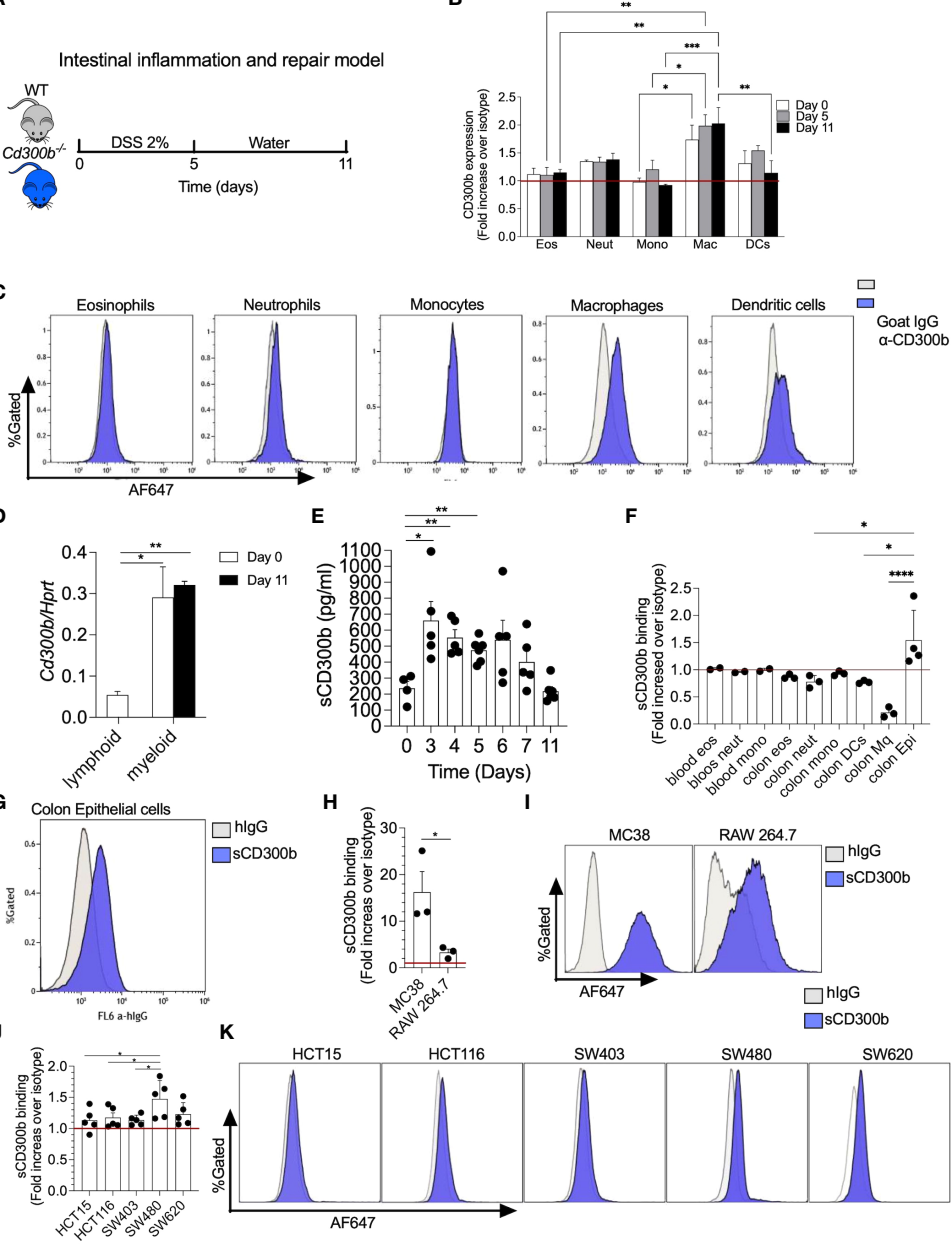
CD300b-Fc binds mouse and human colonic epithelial cells. Schematic representative of intestinal inflammation and repair model is shown **(A)**. Briefly, wild type (WT) mice were treated with 2% dextran sodium sulphate (DSS) for five days to induce colon inflammation. Subsequently, the mice underwent a repair stage by cessation of DSS treatment day 11. Mice were euthanized on days 0, 5, and 11 and the expression of CD300b on the cell surface of colonic eosinophils (Eos), neutrophils (Neut), monocytes (Mono), macrophages (Mac), and dendritic cells (DCs) was determined by flow cytometry **(B)**. Representative histogram plots demonstrating the expression of CD300b in different immune cells is presented **(C)**. Lymphoid and myeloid cells were sorted from the colon and the expression of CD300b was determined by quantitative PCR analysis **(D)**. Punch biopsies from colonic tissue were obtained at the indicated time points and soluble CD300b (sCD300b) was quantified by ELISA **(E)**. Single cell suspensions were obtained from the colon of WT mice **(F, G)** and incubated with CD300b-Fc or hIgG (Isotype control) followed by AF647 conjugated anti-hIgG. Binding of CD300b-Fc fusion protein to the cells was determined by flow cytometry **(F, G)**. MC38 and Raw 264.7 cells **(H, I)**, as well as HCT15, HCT116, SW403, SW480 and SW620 human epithelial ell lines **(J, K)** were incubated with CD300b-Fc or hIgG (Isotype control) followed by AF647 conjugated anti-hIgG. Binding of CD300b was determined by flow cytometry. Data are from n≥4 **(B, D)**, Each dot represents a mouse **(E, F)**, In **(H, J)** each dot represents the average of triplicates. *p<0.05, **p<0.01, ***p<0.001, ****p<0.0001.

Given the paucity of data regarding CD300b in colitis, we first aimed to characterize the cellular source of CD300b and sCD300b during colitis. To this end, DSS was given to WT and *Cd300b^-/-^* mice in their drinking water for 5 days. Thereafter, the DSS was replaced by regular drinking water for an additional 6 days to allow DSS-induced inflammation to subside (**Figure 1A**). Since, CD300b is mainly expressed on myeloid cell lineage we quantitated the expression level membrane CD300b in macrophages, dendritic cells, neutrophils, monocytes, and eosinophils throughout the model (**Figure 1B**, see gating strategy in **Supplemental Figure 1**). Macrophages, DCs and neutrophils displayed the highest level of CD300b expression whereas eosinophils and monocytes displayed little to no CD300b expression (**Figures 1B, C**). Cell surface expression of CD300b was readily detected on days 0, 5 and 11, and remained stable with little to no up or down regulation. Stable expression of CD300b on the surface of colonic CD11b^+^ myeloid cells was also confirmed by quantitative PCR analysis (**Figure 1D**).

Membrane-bound CD300b has been shown to be cleaved in settings of innate immune driven-induced inflammation by LPS. Since DSS-induced colitis initiates a robust innate immune response, we hypothesized that the expression of sCD300b will be increased following induction of colitis. To assess this, colonic “punch biopsies” were obtained from the colons of WT mice (days 0-11) and were incubated for twenty-four hours. Subsequently, expression of sCD300b was determined in the supernatants by ELISA. sCD300b was detectable under baseline conditions (day 0) and its levels were significantly increased on days 3, 4, and 5. Starting on day 6 the expression of sCD300b gradually decreased and by day 11 the levels of scD300b were comparable to those found under baseline conditions (**Figure 1E**).

### CD300b binds mouse and human colonic epithelial cells

The presence and induction of sCD300b following colitis suggests that sCD300b is capable of binding target cells in the colon. To determine which cells can possibly interact with sCD300b, colonic single cell suspensions were obtained, incubated with IgG1 CD300b-Fc fusion protein (as a surrogate for sCD300b) and binding was determined by flow cytometry. Colonic epithelial cells were the only cells that bound to CD300b-Fc fusion (**Figures 1F, G**). The ability of CD300b-Fc fusion protein to bind colonic epithelial cells was further confirmed using a colonic epithelial cell line (i.e., MC38 cells) and macrophage cell line (RAW264.7 cells). Although both cell lines were capable of binding CD300b-Fc fusion protein, MC38 cells bound to CD300b-Fc fusion protein to a higher extent (Fold increase of 16 *vs*. 3.5, in comparison with RAW264.7 cells) (**Figures 1H, I**). Notably, binding of CD300b-Fc was dose dependent on both cell lines (**Supplementary Figure 2**). To determine whether human epithelial cells can bind sCD300b as well, five different human colonic epithelial cell lines (i.e., HCT15, HCT116, SW403, SW480, and SW620) were incubated with CD300b-Fc fusion protein and control antibody. Human colonic epithelial cells bound to CD300b-Fc fusion protein in varying degrees. While SW480 and SW620 cells displayed relatively high levels of binding, HCT15, HCT116 and SW403 showed lower binding capacity (**Figures 1J, K**).

**Figure 2:**
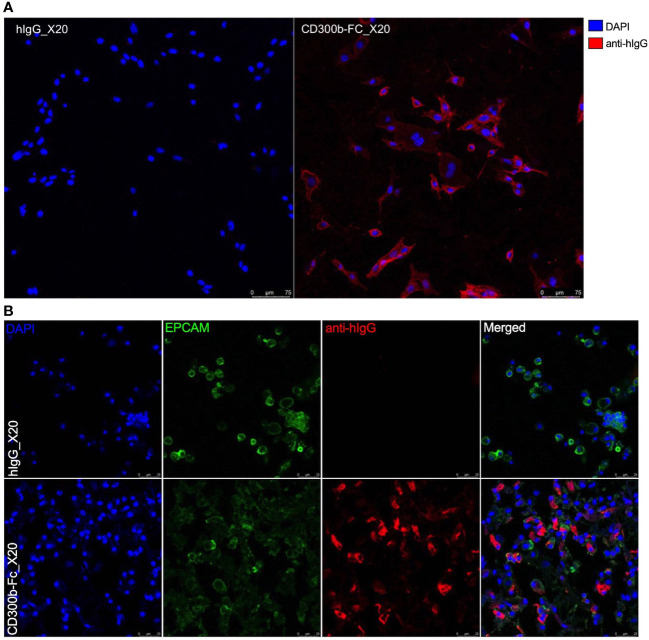
Immunofluorescence of CD300b-Fc colonic epithelial cell binding. Mouse MC38 epithelial cells **(A)** or primary colonic epithelial cells **(B)** were incubated with hIgG1 or CD300b-Fc IgG1 fusion protein. Subsequently, the cells were incubated with donkey anti hIgG conjugated to Alexaflour 647. Epithelial cells were stained with nuclear staining (DAPI, **A, B**) and primary colonic epithelial cells were identified as EpCAM (EPCAM)^+^ cells **(B)**. Representative photomicrographs are shown.

Binding of sCD300b to mouse MC38 cells (**Figure 2A**) and primary colonic epithelial cells (**Figure 2B**) was further confirmed by means of immunofluorescence.

### *Cd300b^-/-^* mice display defects in resolving DSS-induced inflammation

**Figure 3:**
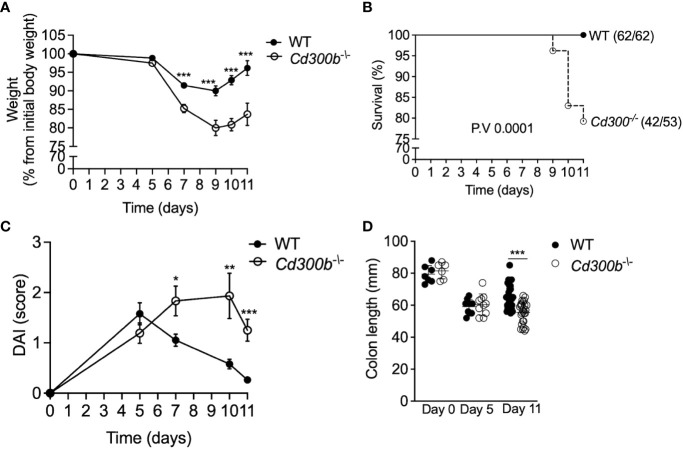
*Cd300b^-/-^* mice display defects in resolving DSS-induced inflammation. WT and *Cd300b^-/-^* mice were subjected to 2% DSS in their drinking water for five days followed by six days of DSS-cessation. Mice were analyzed for weight loss **(A)**, survival **(B)**, clinical disease activity index (DAI) **(C)**, and colon length **(D)**. Graphs **(A, C)** are representative of n=4 experiments conducted with 13-16 mice. In **(B)** numbers in parentheses represent the ratio between the total amount of dead mice to total mice used in all experiments. In **(D)**, Each circle represents one mouse. *p<0.05, **p<0.01, ***p<0.001.

To define the role of CD300b in colonic inflammation WT and *Cd300b^-/-^* mice were subjected to experimental colitis as described in **Figure 1A**. *Cd300b^-/-^* mice displayed significantly increased weight loss, which peaked by day 9 (**Figure 3A**). By day 11, WT mice gradually gained weight and nearly reached their initial weight. In contrast, *Cd300b^-/-^* mice displayed markedly reduced weight even by day 11. Furthermore, starting on day 9 (**Figure 3B**), DSS-treated *Cd300^-/-^* mice begun dying and by day 11, ~21% of the DSS-treated *Cd300b^-/-^* mice died or met humane endpoint criteria. No death was observed in DSS-treated WT mice (**Figure 3B**). Macroscopical examination of the disease activity index (DAI) score, which comprises of rectal bleeding, diarrhea and posture revealed that WT mice displayed their maximal DAI score at day 5. Following day 5, WT mice were capable of gradually returning to their baseline DAI score. On day 5, DSS-treated *Cd300b^-/-^* displayed comparable DAI scores to DSS-treated WT mice. Nonetheless, their DAI gradually increased and peaked by day 10 (**Figure 3C**). Finally, by day 11, the colons of DSS-treated *Cd300b^-/-^* mice were significantly shorter than those of WT mice (**Figure 3D**).

### Increased colonic inflammation following DSS cessation in *Cd300b* ^-/-^ mice is associated with increased histopathology

**Figure 4:**
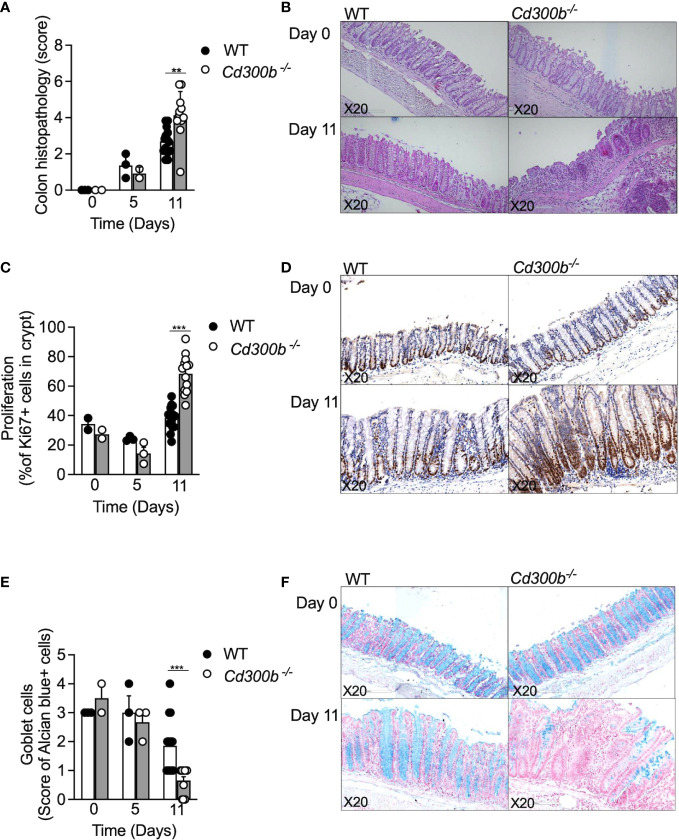
Increased colonic inflammation following DSS cessation in *Cd300b* ^-/-^ mice is associated with increased histopathology. WT and *Cd300b^-\-^* mice were subjected to 2% DSS in their drinking water for five days followed by six days of DSS-cessation and water administration. At days 0,5, and 11 the mice were euthanized, and their colons were obtained for histopathological evaluation. Histopathological score **(A)** and representative photomicrographs of H&E are shown **(B)**. The percentage of proliferating epithelial cells as determined by anti-Ki67 stain **(C)** and representative photomicrographs are shown **(D)**. Quantitation of colonic goblet cells as determined by Alcian blue staining **(E)** and representative photomicrographs are shown **(F)**. Each dot represents one mouse. **p<0.01, ***p<0.001.

Next, colonic histopathology was assessed in WT and *Cd300b^-/-^* mice by subjecting colonic biopsies to hematoxylin and eosin (H&E) staining, anti-Ki67 staining for assessment of epithelial cell proliferation, and alcian blue to quantify goblet cells. Consistent with our macroscopical findings (**Figure 3**), no difference was observed between DSS-treated WT and DSS-treated *Cd300b^-/-^* mice on day 5. On day 11 however, the colons of DSS-treated *Cd300b^-/-^* mice displayed marked epithelial cell erosion, oedema, and cellular infiltration in comparison with WT mice (**Figures 4A, B**). In addition, on day 11, DSS-treated *Cd300b^-/-^* mice displayed increased epithelial cell proliferation in comparison with WT mice, which had comparable epithelial cell proliferation as baseline (day 0, **Figures 4C, D**). Moreover, on day 11 DSS-treated *Cd300b^-/-^* mice displayed reduced numbers of goblet cells in comparison with WT mice, which begun to restore the composition of their colonic goblet cells (**Figures 4E, F**). These data suggest increased inflammation and delayed repair in the colons of DSS-treated *Cd300b^-/-^* mice.

### CD300b regulates colonic cellular infiltration and cytokine production

**Figure 5:**
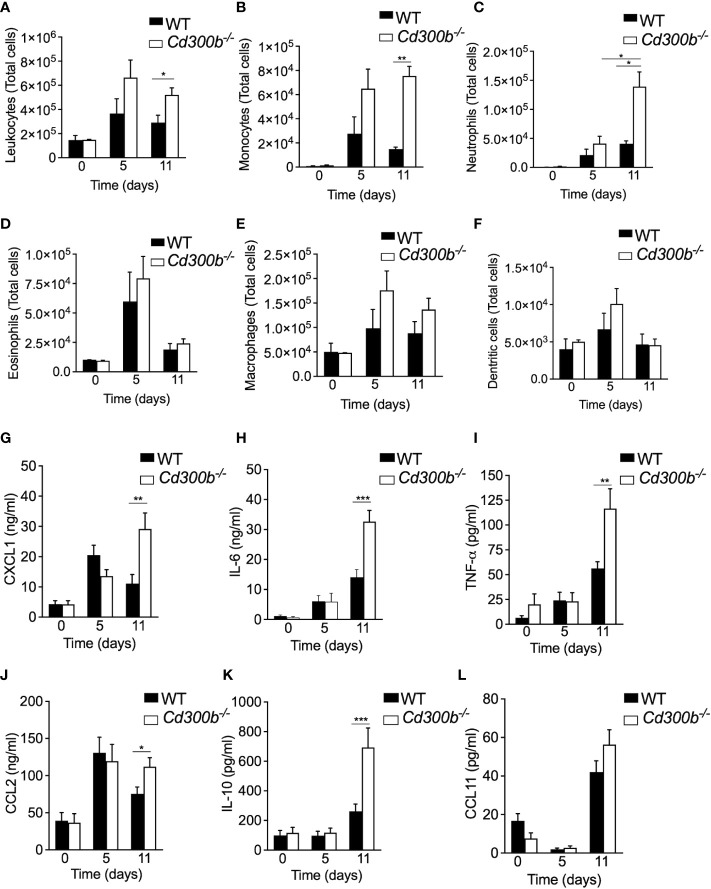
CD300b regulates colonic cellular infiltration and cytokine production. WT and *Cd300b^-\-^* mice were subjected to 2% DSS in their drinking water for five days followed by six days of DSS-cessation and water administration. On days 0,5 and 11, the mice were euthanized, the colon obtained, enzymatically digested and the number of immune cells in the colon was analysed by flow cytometry **(A–F)**. Data are from n=3 experiments with 8-10 mice. *p<0.05, **p<0.01, ***p<0.001. Colonic “punch biopsies” were obtained on days 0, 5 and 11, and incubated for 24 hours in culture medium. Levels of CXCL1 **(G)**, IL-6 **(H)**, TNF-α **(I)**, CCL2 **(J)**, IL-10 **(K)**, AND CCL11 **(L)** in the culture supernatants are shown. Data are from n ≥ 10 mice. *p<0.05, **p<0.01, ***p<0.001.

Increased DAI and histopathology in the colons of DSS-treated *Cd300b^-/-^* mice suggested that *CD300b^-/-^* mice will display increased infiltration of immune cells as well. Indeed, DSS-treated *Cd300b^-/-^* mice exhibited increased influx of leukocytes into the colon, which was evident by day 5 and statistically significant by day 11 (**Figure 5A**). Increased leukocytes in DSS-treated *Cd300b* were mostly due to increased levels of monocytes and neutrophils, which were markedly increased (**Figures 5B, C**). No significant alterations were observed on days 0, 5, and 11 in eosinophil, macrophage, and dendritic cell populations (**Figures 5D–F**). Analysis of cytokine/chemokine expression in the supernatants of colonic punch biopsies revealed increased levels of CXCL1, IL-6, TNF-α, CCL2, IL-10 but not CCL11, EGF, VEGF and IL-33 in DSS-treated *Cd300b^-/-^* mice in comparison with WT mice (**Figures 5G–L** and **Supplementary Figure 3**). Consistent with our previous data, alterations in cytokine/chemokine expression were observed on day 11 but not at day 5 (**Figures 5G–L**, respectively).

### CD300b-induced clearance of apoptotic cells does not regulate colonic inflammation

**Figure 6:**
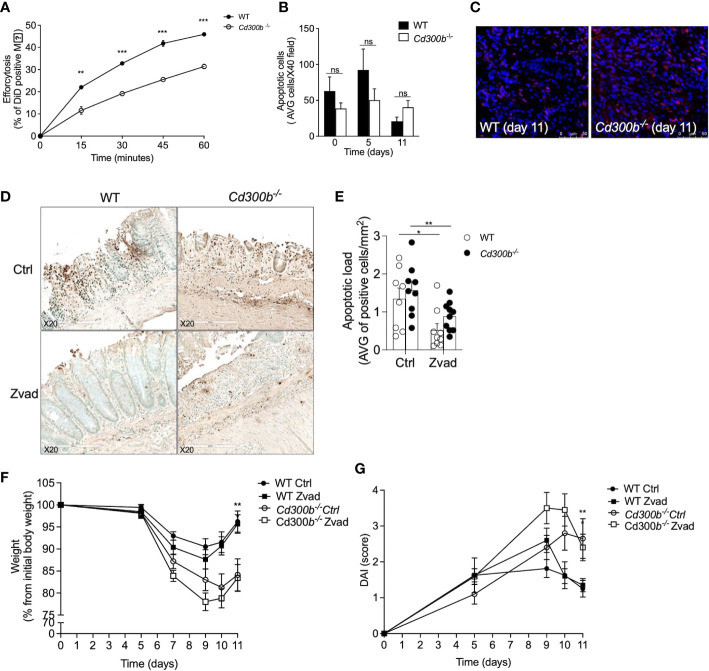
CD300b-induced clearance of apoptotic cells does not regulate colonic inflammation. WT and *Cd300b^-\-^* bone marrow-derived macrophages (BMDMs) were stained with CFSE and incubated with DiD-labeled apoptotic MC38 cells (1:5 ratios, respectively) for the indicated time points. The percentage of engulfed dead cells (Efferocytosis) was determined by flow cytometry **(A)**. WT and *Cd300b^-\-^* mice were subjected to 2% DSS in their drinking water for five days followed by six days of DSS-cessation and water administration. On days 0, 5, and 11 the mice were euthanized, their colons were obtained and stained for the presence of apoptotic cells (AC) using TUNEL staining **(B)**. Representative photomicrographs (magnification X40) of TUNEL staining are shown **(C)**. Mice were injected with zVAD or vehicle (Ctrl) every other starting on day 5. Representative photomicrographs and quantitative analysis of TUNEL^+^ cells are shown **(D, E)**. Thereafter, the mice were monitored for changes in weight **(F)** and assessed for disease activity index (DAI) **(G)**. Data are representative of n=3 experiments **(A–C)**. In **(D, E)** data is from n=2 experiments with n ≥ 8 mice per group. ns, non significant, *p<0.05, **p<0.01, ***p<0.0 01.

Increased pathology in DSS-treated *Cd300b^-/-^* mice suggest an anti-inflammatory role for CD300b. Since CD300b can bind PtdSer and regulate efferocytosis, we hypothesized that the absence of CD300b impairs resolution of inflammation due to inability to clear apoptotic cells. To test this hypothesis, we were first interested to assess the role of CD300b in efferocytosis *in vitro*. For this, WT and *Cd300b^-/-^* bone marrow-derived macrophages (BMDMs) were generated and incubated with apoptotic cells. CD300b was required for proper efferocytosis *in vitro* (**Figure 6A**). Next, we assessed whether *Cd300b^-/-^* mice displayed increased numbers of apoptotic cells in their colons. No differences were observed between the apoptotic cell numbers in control or DSS-treated WT and *Cd300b^-/-^* mice (**Figures 6B, C**). To functionally determine whether increased pathology in DSS-treated *Cd300b^-/-^* mice was due to defects in efferocytosis, the pan-caspase inhibitor zVAD was injected to WT and *Cd300b^-/-^* mice starting from day 5 as described (21). Assessment of apoptotic cells using TUNEL staining demonstrated decreased numbers of apoptotic cells in the colons of WT and *Cd300b^-/-^* mice in comparison with control treated mice (**Figures 6D, E**). Notably, there was no difference in the levels of apoptotic cells between WT and *Cd300b^-/-^* mice (**Figures 6D, E**). Despite administration of zVAD, DSS-treated *Cd300b^-/-^* mice still presented increased weight loss and DAI score (**Figures 6F, G**). This data suggests that CD300b-induced clearance of apoptotic cells is not the underlying mechanism for increased pathology.

### sCD300b promotes wound healing and regulates epithelial cell motility

**Figure 7:**
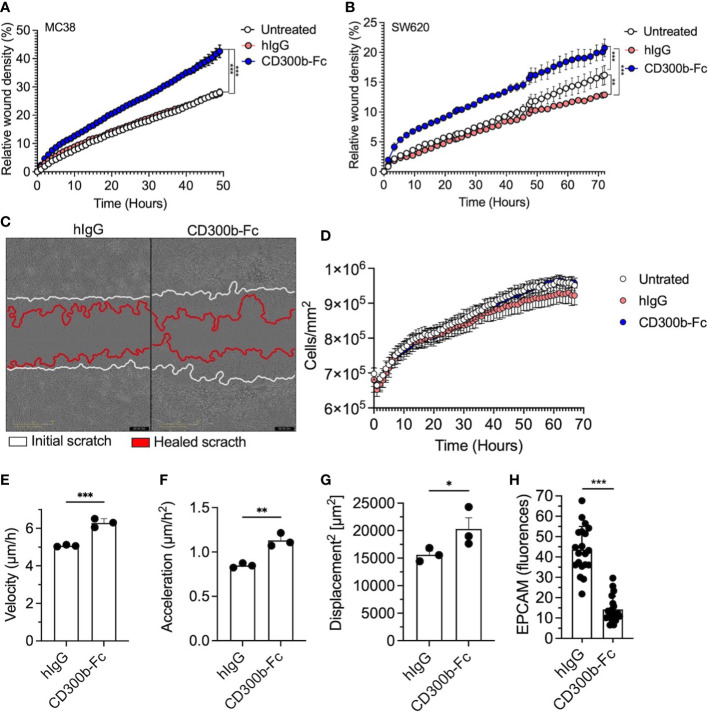
sCD300b promotes wound healing and regulates epithelial cell motility. MC38 and SW620 cells were incubated with CD300b-Fc fusion protein or hIgG and a scratch wound assay was performed. Relative wound density **(A, B)** and a representative photomicrograph of MC38-treated cells is shown **(C)**. Epithelial cell proliferation **(D)**, velocity **(E)**, acceleration **(F)** and displacement **(G)** were calculated from at least 100 single epithelial cells. Surface expression of EpCAM was determined by immunofluorescence **(H)**. Data are from n=3, *p<0.05, **p<0.01, ***p<0.001.

The increase in sCD300b and its ability to specifically bind epithelial cells raised the hypothesis that sCD300b can directly promote mucosal healing by regulating epithelial cell activities. To test this hypothesis mouse MC38 cells were incubated with CD300b-Fc fusion protein or isotype control (hIgG) and a scratch assay was performed. Mouse CD300b-Fc fusion protein-treated cells displayed increased wound closure in comparison with control treated cells (**Figure 7A**). Similarly, human CD300b-Fc fusion protein induced increased wound healing of the human colonic epithelial cell line SW620 (**Figures 7B, C**). Importantly, this effect was time dependent since it occurred only after over-night pre-treatment of colonic epithelial cells with CD300b-Fc fusion protein and not following shorter incubation periods (**Supplementary Figure 4**). Treatment of mouse epithelial cells with CD300b-Fc fusion protein had no effect on epithelial cellular proliferation or efferocytosis (**Figure 7D** and data not shown). Thus, we hypothesized that sCD300b regulates epithelial cell motility. Using a single-cell resolution based morphokinetic analysis (20) of CD300b-Fc fusion protein treated cells, demonstrated increased epithelial cell velocity, acceleration, and displacement in CD300b-Fc fusion protein treated cells (**Figures 7E–G**). In support of these data, treatment of primary colonic epithelial cells with CD300b-Fc fusion protein resulted in decreased surface EpCAM expression (**Figure 7H**).

## Discussion

Inflammatory responses in the gastrointestinal (GI) tract are tightly regulated by a large network signal that provides a fundamental basis for intestinal homeostasis (22). The critical involvement of this regulation is apparent during multiple diseases (e.g., Chron’s disease and ulcerative colitis) where colonic inflammation drives disease pathogenesis and is a main therapeutic target (2). Indeed, current medications targeting inflammatory pathways provide the first line of therapy in IBD and are capable of inducing disease remission in many patients by dampening inflammation and initiating mucosal healing (2, 23). Consequently, mucosal healing and GI repair have emerged as a key treatment goal in IBD that predicts decreased need for corticosteroids, decreased hospitalization rates, sustained clinical remission, decreased colectomy and bowel resection and finally, decreased risk for colorectal cancer (3). Thus, molecular mechanisms that limit GI inflammation and enhance resolution have significant therapeutic potential. The CD300-family of receptors draw much attention because their genetic loci is one of only a few loci in the genome that is under strong positive evolutionary selection (6), likely indicating the importance of these receptors in immune regulation. Furthermore, others and we, have recently linked the expression and activity of these receptors to colonic inflammation (13, 24). Herein, we provide several lines of evidence for a central and distinct role for CD300b in regulating colonic inflammation likely *via* sCD300b. We demonstrate that sCD300 is increased in the colons of mice that were treated with DSS. We further establish that CD300b can bind mouse and human epithelial cells. We demonstrate that CD300b is required for optimal mucosal healing following induction of acute inflammation. Indeed, *Cd300b^-/-^* mice displayed increased weight loss and disease activity index, which was accompanied by increased colonic histopathology, increased infiltration of inflammatory cells and expression of multiple pro-inflammatory cytokines. Finally, we show that CD300b promotes epithelial cell migration and wound healing. Collectively, these data highlight a key role for CD300b in colonic inflammation and repair processes and suggest that CD300b may be a future therapeutic target in inflammatory GI diseases.

Recent data including our own link the expression and activity of various CD300-family members with intestinal inflammation (4, 25). In support of these findings, we demonstrate that CD300b is expressed on the surface of various immune cells in the GI tract. Unlike the expression of CD300f, which was dynamically regulated during DSS-induced colitis, the expression of CD300b on the surface of immune cells remained stable throughout our experimental protocol. Interestingly, expression of sCD300b was increased. These findings are consistent with previous reports demonstrating that soluble CD300b levels were increased in a model of LPS-induced inflammation. Increased expression of sCD300b is not surprising since the expression of various CD300-family members has been shown to regulated by various environmental factors including cytokines and innate immune components (26–28). Furthermore, mouse CD300b has been shown to be cleaved by neutrophil matrix metalloproteinases (15), which may be increased in DSS-induced colitis and are elevated in human IBD (29). Increased expression of sCD300b was observed as early as 3 days following treatment with DSS and remained stale up to day 7. Interestingly, *Cd300b^-/-^* mice displayed increased pathology on day 11 when sCD300b expression was decreased. This could be explained at least in part by our *in vitro* data suggesting a time-dependent effect for sCD300b on epithelial cells. Alternatively, we have identified that upon incubation with epithelial cells, sCD300b is internalized (data not shown). Thus, decreased expression could be due to internalization and subsequent cellular activation. Future studies should determine the mechanistic contribution of sCD300b internalization.

Using CD300b-Fc fusion protein we show that CD300b and sCD300b can potentially bind colonic epithelial cells. Even though we show binding of Raw 264.7 cells to CD300b-Fc, we could not detect any binding in primary colonic macrophages. This finding is of specific interest since recently, sCD300b was proposed to amplify macrophage-induced inflammatory signaling *via* binding to a yet unknown receptor on their surface (15). The difference between our findings and previous reports suggests that exposure to environmental endotoxins and/or different microbiota communities that are present in the GI tract may modulate the expression of ligands for CD300b.

CD300 family receptors have been shown to bind various ligands that are either expressed by bacteria (30), yeast (31) or are associated with cell death (4, 7). Therefore, we selected to assess the role of CD300b using the DSS model as it induces epithelial cell shedding, cell death (32, 33), and exposes foreign antigens some of which may bind and activate CD300b. The importance of CD300 receptor-ligands interactions in the resolution of immune responses has been documented in various settings especially in the regulation of allergic, autoimmune and innate immune responses (4, 8, 11, 12, 31, 34–36). For example, deficiency in *Cd300f* leads to enhanced antigen processing by dendritic cells and subsequent priming of T cells. Consequently, this leads to increased expansion of memory T cells, which predispose *CD300f^-/-^* mice to develop a systemic lupus erythematosus-like autoimmune disease when exposed to an overload of apoptotic cells, or an exacerbated autoimmunity when combined with FcγRIIB deficiency (8). In addition, the absence of CD300a led to increased allergic responses in a mouse model of allergic peritonitis. *CD300a^-/-^* mice displayed a rapid increase in their inflammatory cell infiltrates and in their tryptase content in the peritoneal cavity compared with wild type (37). Importantly, the resolution process in *Cd300a^-/-^* was altered as well. Defects in the ability to resolve colonic inflammation were also observed in *CD300f^-/-^* mice that expressed lower levels of ALX/FPR2 receptor on peritoneal cells and had higher levels of LXA_4_ in their peritoneal lavage fluid (37). In these settings deficiency of *CD300f* led to defects in dendritic cell functions that were associated with abnormal accumulation of apoptotic cells in the GI tract (34). Mechanistically, *CD300f^-/-^* dendritic cells displayed increased efferocytosis, which induced excessive TNF-α secretion, which in turn, initiated IFN-γ overproduction by colonic T cells, leading to prolonged gut inflammation (34). Similarly, we demonstrate increased expression of various proinflammatory cytokines such as TNF-α, IL-6, and CXCL1 in the colons of DSS-treated *Cd300b^-/-^* mice. We also demonstrate increased expression of the anti-inflammatory cytokine IL-10. Co-expression and/or production of pro- and anti-inflammatory cytokines in inflammation settings is likely since every inflammatory condition can induce simultaneous production of opposing inflammatory signals. For example, stimulation of macrophages with LPS, results in increased secretion of IL-6 and TNF-α but also increases their ability to secrete IL-10 (17). Thus, the “net” effect of an inflammatory response *in vivo* is driven from complex interactions between pro- and anti-inflammatory forces.

Our findings suggest that the role of CD300b in colitis is likely not mediated *via* its ability to mediate and/or regulate efferocytosis. This was evident by the fact that inhibition of apoptosis in WT and *Cd300b^-/-^* mice did not alter the increased susceptibility of *Cd300b^-/-^* mice to DSS-induced colitis. This conclusion should be taken with caution since even though zVAD treatment significantly reduced the levels of apoptotic cells, they were not eliminated. In addition, we cannot rule out the contribution of other forms of cell death such as necroptosis (38). Certainly, zVAD treatment of L929 cells was shown to induce necroptosis *via* autocrine secretion of TNF-α and activation of the transcription factor AP-1 (39).

A possible explanation for increased pathology and defects in resolution in the absence of CD300b may be by binding of sCD300b to colonic epithelial cells and direct induction of epithelial cell responses by sCD300b. This hypothesis is corroborated by the fact that mouse and human colonic epithelial cells bind CD300b-Fc, which serves as a surrogate for sCD300b. Binding of CD300b-Fc fusion protein to the surface of primary colonic epithelial cells was associated with decreased expression of the adhesion molecule EpCAM (40). Decreased expression of adhesion molecules in epithelial cells could lead to increased motility and/or migration. While we could not directly examine the role of sCD300b in promoting epithelial cell migration, incubation of epithelial cells with CD300b-FC fusion protein increased various aspects of epithelial cell motility including velocity, acceleration, and displacement. These data suggest that besides the activities of CD300 family members in resolution of inflammation *via* efferocytosis, they may directly induce resolution and mucosal healing *via* interactions with epithelial cells.

Taken together, our results define a key requirement for CD300b in GI inflammation and repair processes likely by interactions between sCD300b and a yet unknown ligand on colonic epithelial cells. These interactions are required for induction of optimal resolution and healing following acute inflammation. Thus, our study highlights CD300b as a potential therapeutic target in multiple inflammatory GI diseases.

## Data availability statement

The original contributions presented in the study are included in the article/**Supplementary Materials**. Further inquiries can be directed to the corresponding author.

## Ethics statement

The animal study was reviewed and approved by Tel-Aviv University Institutional Animal Care Unit (Ethical approval #034_b14214_48, #034_b13792_20).

## Author contributions

SA and AM- conception and/or design of the work. SA, HK, MI, HR, AD, IH, SG-T, YG, DK-A, PR- Data Collection.AD, HK, IT, AM- Data analysis and interpretation. DK-A- Critical reagents for the study. SA, AM- Drafting the article. SA, AM - Revision of the article. AM- Final approval of the version to be published. All authors contributed to the article and approved the submitted version.
