# Supplementary figures and images for: CD300b regulates intestinal inflammation and promotes repair in colitis
Source: Front Immunol. 2023 Mar 22;14:1050245. doi: 10.3389/fimmu.2023.1050245 (PMC10073762; doi:10.3389/fimmu.2023.1050245)

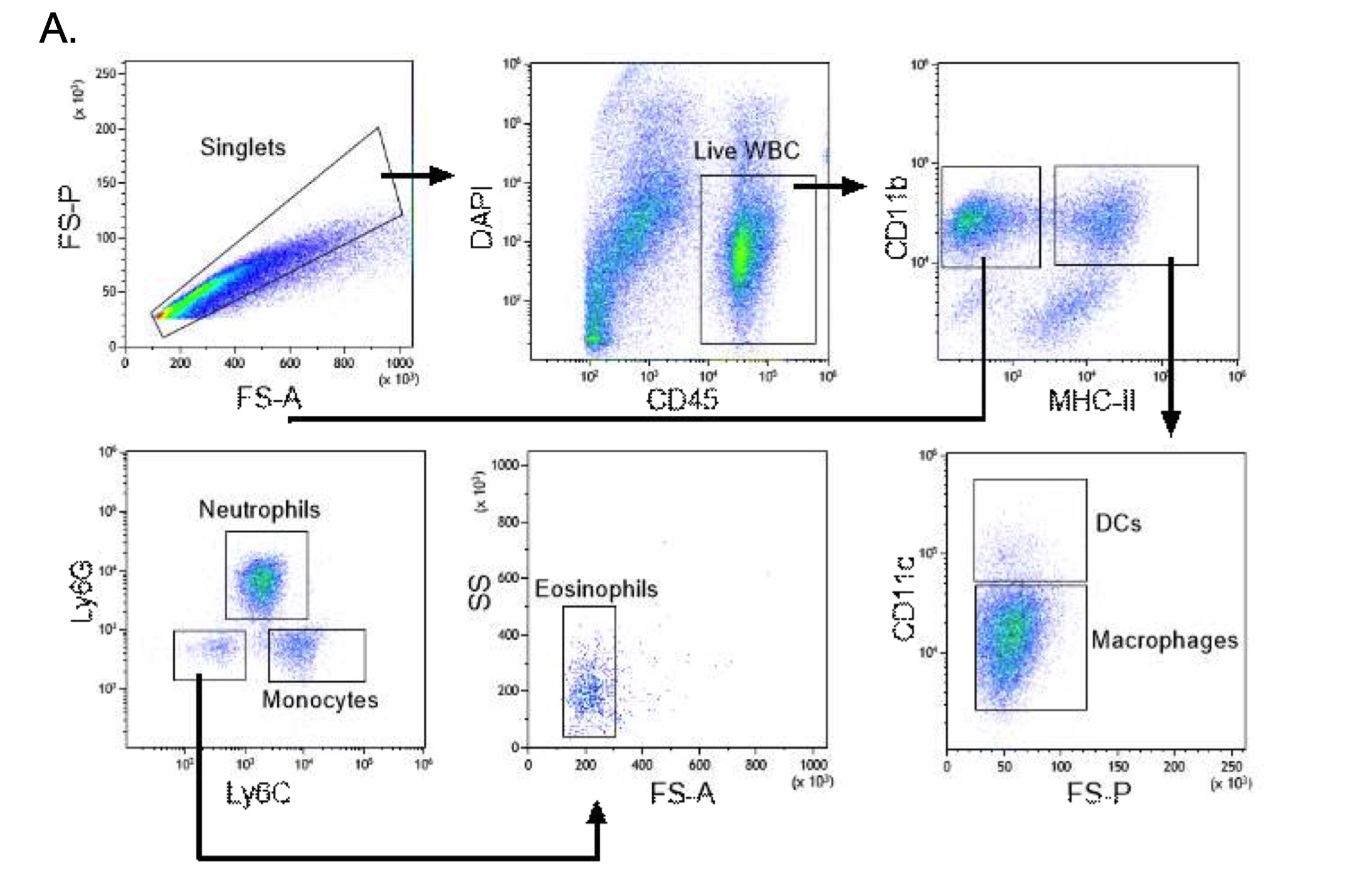

Supplement: Supplementary file 1 [file Image_1.jpeg]

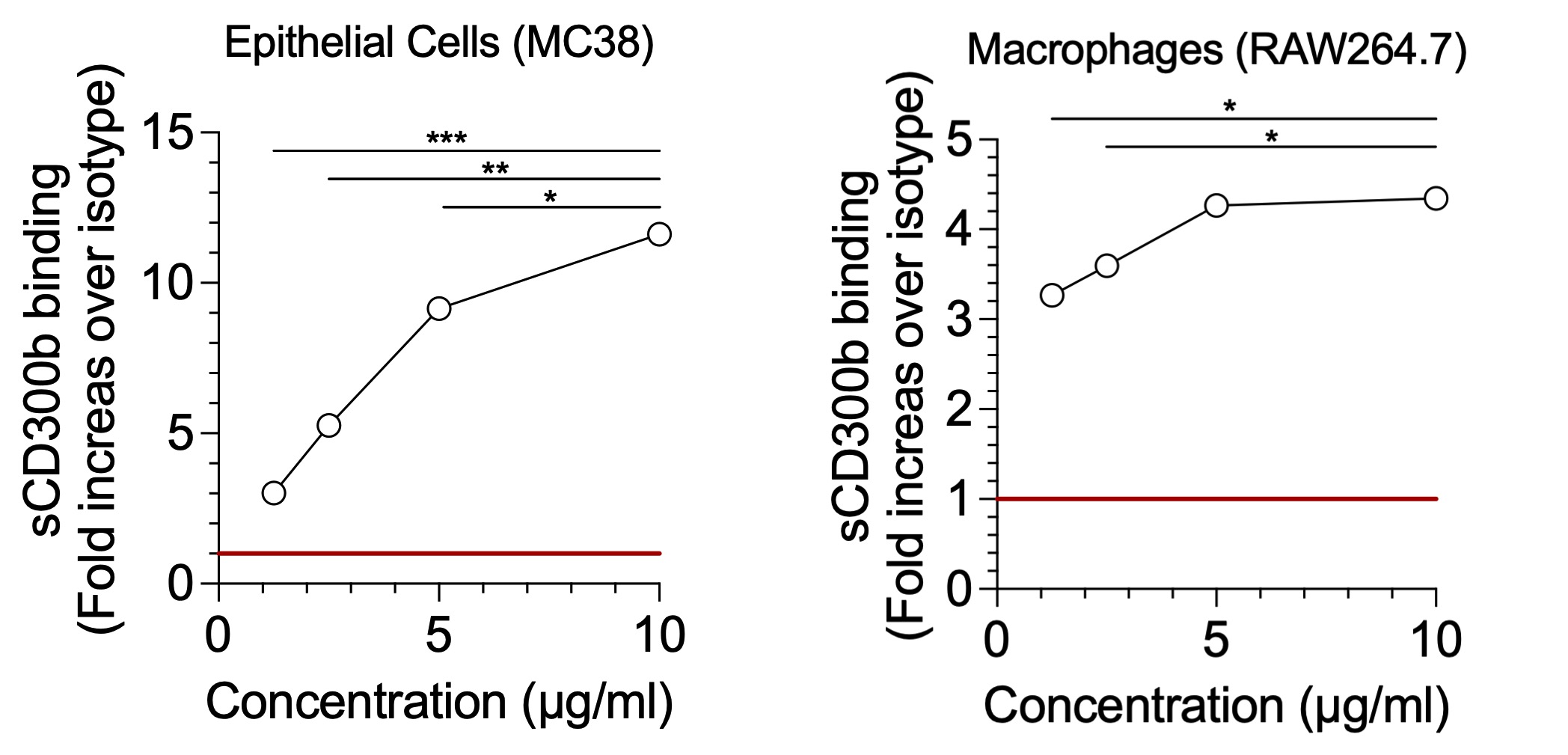

Supplement: Supplementary file 2 [file Image_2.jpeg]

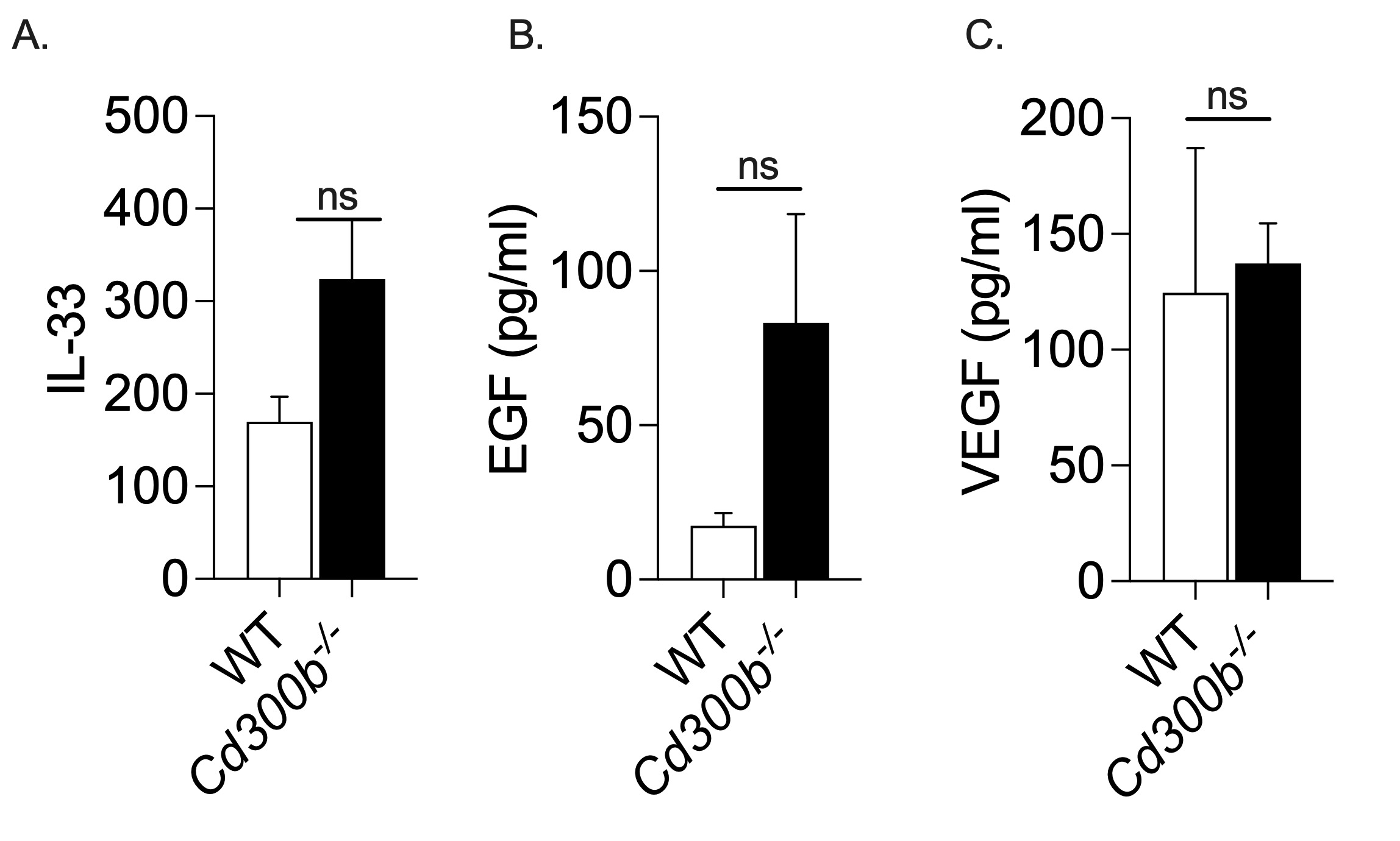

Supplement: Supplementary file 3 [file Image_3.jpeg]

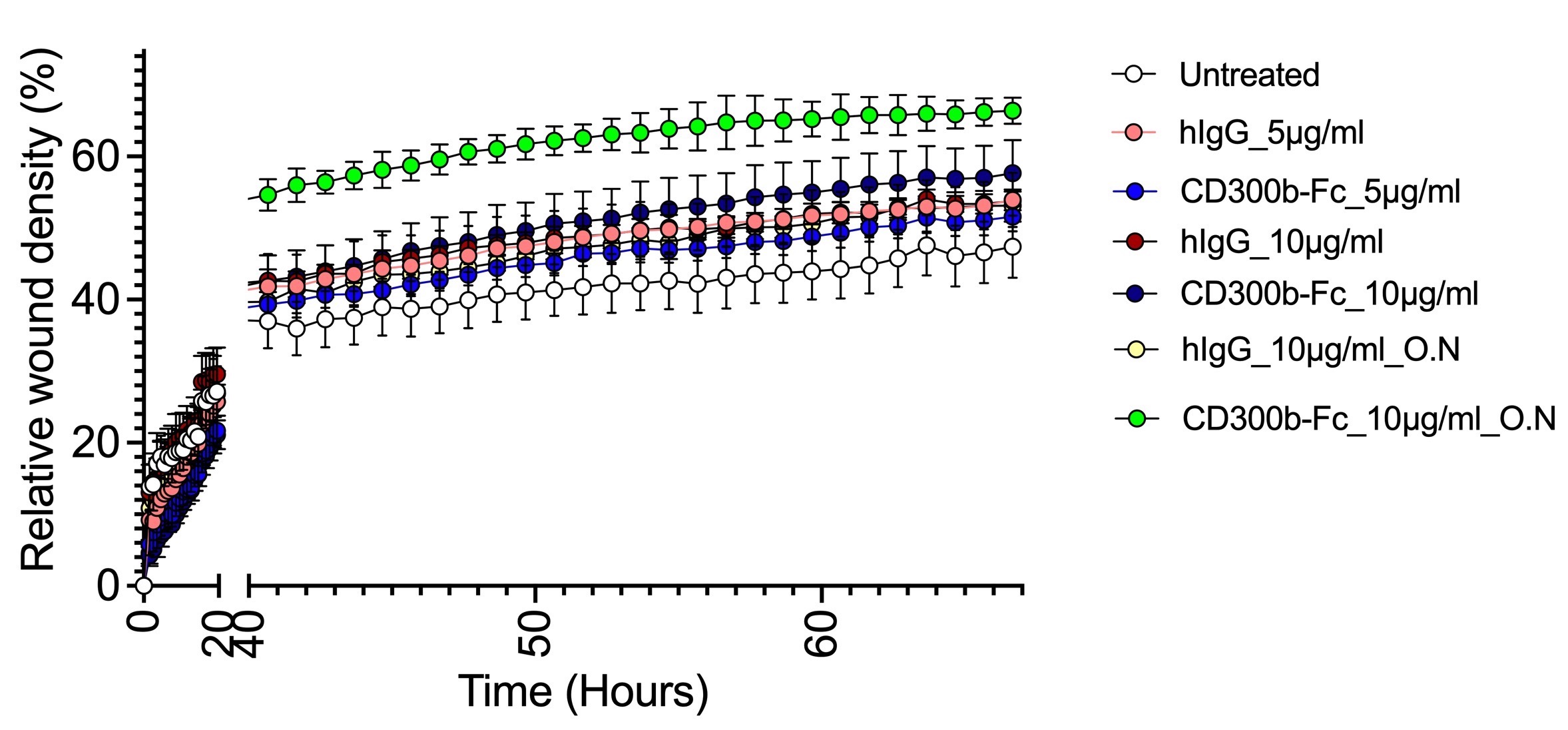

Supplement: Supplementary file 4 [file Image_4.jpeg]
